# Myosin Light Chain Kinase Expression Induced via Tumor Necrosis Factor Receptor 2 Signaling in the Epithelial Cells Regulates the Development of Colitis-Associated Carcinogenesis

**DOI:** 10.1371/journal.pone.0088369

**Published:** 2014-02-10

**Authors:** Masahiro Suzuki, Takashi Nagaishi, Motomi Yamazaki, Michio Onizawa, Taro Watabe, Yuriko Sakamaki, Shizuko Ichinose, Mamoru Totsuka, Shigeru Oshima, Ryuichi Okamoto, Motoyuki Shimonaka, Hideo Yagita, Tetsuya Nakamura, Mamoru Watanabe

**Affiliations:** 1 Department of Gastroenterology, Graduate School of Medical Science, Tokyo Medical and Dental University, Tokyo, Japan; 2 Department of Obstetrics and Gynecology, Nihon University School of Medicine, Tokyo, Japan; 3 Research Center for Medical and Dental Sciences, Tokyo Medical and Dental University, Tokyo, Japan; 4 Department of Applied Biological Chemistry, The University of Tokyo, Tokyo, Japan; 5 Department of Chemistry, Tokyo University of Science, Tokyo, Japan; 6 Department of Immunology, Juntendo University School of Medicine, Tokyo, Japan; Massachusetts General Hospital, United States of America

## Abstract

It has been suggested that prolonged inflammatory bowel diseases (IBD) may lead to colitis-associated carcinogenesis (CAC). We previously observed that the NF-κB activation in colonic epithelial cells is associated with increased tumor necrosis factor receptor 2 (TNFR2) expression in CAC development. However, the mechanism by which epithelial NF-κB activation leading to CAC is still unclear. Myosin light chain kinase (MLCK) has been reported to be responsible for the epithelial permeability associated with TNF signaling. Therefore we focused on the role of MLCK expression via TNFR2 signaling on CAC development. Pro-tumorigenic cytokines such as IL-1β, IL-6 and MIP-2 production as well as INF-γ and TNF production at the lamina propria were increased in the setting of colitis, and further in tumor tissues in associations with up-regulated TNFR2 and MLCK expressions in the epithelial cells of a CAC model. The up-regulated MLCK expression was observed in TNF-stimulated colonic epithelial cells in a dose-dependent fashion in association with up-regulation of TNFR2. Silencing TNFR2, but not TNFR1, resulted in restoration of epithelial tight junction (TJ) associated with decreased MLCK expression. Antibody-mediated blockade of TNF signaling also resulted in restoration of TJ in association with suppressed MLCK expression, and interestingly, similar results were observed with suppressing TNFR2 and MLCK expressions by inhibiting MLCK in the epithelial cells. Silencing of MLCK also resulted in suppressed TNFR2, but not TNFR1, expression, suggesting that the restored TJ leads to reduced TNFR2 signaling. Such suppression of MLCK as well as blockade of TNFR2 signaling resulted in restored TJ, decreased pro-tumorigenic cytokines and reduced CAC development. These results suggest that MLCK may be a potential target for the prevention of IBD-associated tumor development.

## Introduction

Although the pathogenesis of inflammatory bowel disease (IBD), such as Crohn’s disease and ulcerative colitis in humans, still remains unclear, chronic epithelial permeability seems to be one of the mechanisms by which extensive inflammatory factors may be introduced into the irritated intestinal tissues. Therefore, it is believed that induction of mucosal healing is critical in the management of IBD [1]. Furthermore, chronic inflammation is believed to associate with carcinogenesis, and prolonged duration of IBD likely also lead to colitis-associated cancer (CAC) [2], [3], [4].

Previous study had shown that activation of NF-κB in the inflamed tissue is strongly associated with carcinogenesis [5]. In this regard, we have investigated the mechanism of NF-κB activation in the colonic epithelial cells using a murine model of IBD. We have previously reported that increased expression of tumor necrosis factor (TNF) in a murine IBD model is critical for the development of CAC [6]. TNF is a pivotal cytokine associated with the continuous immune dysregulation in the inflamed tissue of IBD [7], [8]. In our previous study, the specific up-regulation of the type 2 receptor for TNF (TNFR2) was also observed in the inflamed intestinal epithelial cells. This observation seems logical since the cytoplasmic domain of TNFR2 can also activate NF-κB pathway, but it lacks association with the death domains (DD) like that of TNFR1. However, the specific role of such NF-κB activation in the inflamed epithelia via TNFR2 signaling in the context of CAC has not been elucidated.

Myosin light chain kinase (MLCK) has also been reported to be expressed in the human intestinal tissue with IBD [9]. MLCK is classically known to be required for the contraction of actomyosin via the phosphorylation of myosin light chain (MLC) [10]. It is also essential to the permeability of epithelial barrier according to in vitro and in vivo studies, and it is associated with the production of pro-inflammatory cytokine, such as TNF, in the inflamed intestinal tissues [9], [11]. In addition, several recent reports have implicated the role of MLCK in animal models of IBD [12], [13], [14]. However, the association between MLCK and CAC development has not been reported. We hypothesized that one of the roles of epithelial NF-κB activation would be the induction of MLCK in the context of IBD. We therefore examined the role of MLCK in the development of IBD-associated carcinogenesis.

## Materials and Methods

### Cell Culture

Murine colonic epithelial cell line, MOC1 [15], which was generated from ‘non-tumor’ colonic epithelia of BALB/c and transformed with SV40 large T antigen, was established by Dr. M. Totsuka (University of Tokyo, Japan) and maintained in RPMI 1640 (Sigma, St. Louis, MO) supplemented with 5% fetal bovine serum, 500 units/ml penicillin, 100 µg/ml streptomycin (Sigma) and 10 µg/ml insulin (Sigma) at 37°C in 5% CO_2_. Cells were seeded at a density of 5×10^4^ cells/ml in 6-well plates 24–36 h prior to the experiments with or without recombinant (r) mouse interferon (IFN)-γ and/or r mouse TNF (Peprotek, London, UK). In some experiments, cells were also incubated in the presence of either blocking anti-mouse TNF monoclonal antibody (mAb) (MP6-XT22, rat IgG1b) (DNAX Research Institute, Palo Alto, CA) [6] or MLCK inhibitor, ML-7 (Sigma).

### Animals

Wild-type female C57BL/6 mice (6–8 wk old) were purchased from Japan Clea (Tokyo, Japan) and maintained under specific pathogen-free conditions in the Animal Care Facility of Tokyo Medical and Dental University (TMDU), Japan. Mice were used between 8–10 weeks of age. All animal experimentations were approved by the Animal Review Board of TMDU and were performed in accordance with institutional guidelines.

### Induction of Chronic Colitis and CAC Models

Mice were randomized by body weight into three groups (n = 5) and given intraperitoneal (i.p.) 10 mg/kg of azoxymethane (AOM, Sigma) at day −7, followed by the administration of three cycles of 2.0% dextran sodium sulfate (DSS, molecular weight 10,000; Yokohama Kokusai Bio, Kanagawa, Japan) for 5 days and regular water for 16 days, and then injected i.p. with either the inhibitor against MLCK, ML-7 (2.0 mg/kg) or the vehicle control (2.0% ethanol) every 12 h from day 63 for 7 days. In some experiments, mice were injected i.p. with either 2.0 mg/kg of ML-7 every 12 h or 50 mg/kg of MP6-XT22, an anti-mouse TNF mAb, weekly starting at the end of first DSS treatment (day 5) (n = 10) and then euthanized at day 70. Mice were euthanized 11 weeks after the first injection of AOM, and colons were removed and immediately flushed with PBS. The isolated colonic epithelial samples from mice were prepared as previously described [16] for assessment of protein and/or mRNA expression in the epithelia.

### Histological Scoring of Colitis and CAC

Tissues from proximal and distal colons were removed for histologic assessment. For this, the tissue samples were fixed in 10% neutral-buffered formalin. Paraffin-embedded sections (5 µm) were stained with hematoxylin and eosin (H-E). The sections were analyzed without prior knowledge of the types of treatments. The histological scoring of colitis was determined according to the previously described system with minor modifications [6], [17]. Briefly, for extent of leukocyte infiltration in tissue layers, 0 points were assigned to normal appearance, or the presence of occasional inflammatory cells in the lamina propria; 1 point to increased numbers of inflammatory cells in the lamina propria; 2 points to confluence of inflammatory cells, extending into the submucosa; and 3 points to transmural extension of the infiltrate. For severity of leukocyte infiltration, 0 points were assigned to none to normal lymphoid aggregates; 1 point to mild infiltration; 2 points to moderate infiltration; 3 points to severe infiltration. For extent of leukocyte infiltration in colon, 0 points were assigned to none to occasional infiltration; 1 point to patchy (focal) infiltration; 2 points to intermediate infiltration; 3 points to diffuse (extensive) infiltration.

For tissue damage, 0 points were assigned to no mucosal damage; 1 point to discrete lymphoepithelial lesions; 2 points to surface mucosal erosion or focal ulceration; and 3 points to extensive mucosal damage and extension into deeper structures of the bowel wall. The cumulative degree of these parameters was calculated as a total histological score ranging from 0 (no changes) to 6 (extensive cell infiltration and tissue damage). For crypt abscess, the assigned points were; 0, to no crypt abscess; 1, to the presence of crypt abscess. For CAC assessment, sections (5 µm) were cut stepwise (200 µm) through the complete block and stained with H-E. Tumor numbers were counted by trained individuals blinded to the treatment group.

### Western Blotting

Western blotting was performed as previously described [18], [19]. Briefly, 10 to 100 µg of nucleic extracts or whole protein lysates from either the stripped epithelial samples or MOC1 cells were separated by 8–15% SDS-PAGE and each protein expressions were analyzed with following primary and secondary Abs: anti-mouse TNFR1 polyclonal Ab (pAb), anti-mouse TNFR2 pAb (R&D Systems, Minneapolis, MN), anti-phosphorylated (p)-p65 mAb at serine 536, anti-p65 pAb, anti-p-IκBα mAb at serine 32/36, anti-IκBα pAb, anti-p-MLC pAb, anti-MLC pAb (Cell Signaling Technology Inc, Beverly, MA), anti-MLCK mAb, anti-β-actin mAb (Sigma), anti-mouse IgG-HRP, anti-rabbit IgG-HRP (GE Healthcare Bio-Sciences, Piscataway, NJ) and anti-goat IgG-HRP (Santa Cruz). Signals were generated with ECL Western Blotting Detection System (GE Healthcare Bio-Sciences).

### Semi-quantitative PCR (q-PCR)

Total cellular RNA was extracted from either whole colonic mucosa, isolated epithelial samples of non-tumor or tumor area removed from AOM/DSS-treated mice, or cultured MOC1 cells with RNA-Bee (Tel-Test, Inc, Friendswood, TX). Five micrograms of total RNA were subjected to reverse transcription using Superscript Reverse Transcriptase kit (Invitrogen, Carlsbad, CA). The cDNA samples were then applied for PCR with the following primer pairs: interleukin (IL)-1β, 5′-TTG ACG GAC CCA AAA GAT-3′ and 5′-GAA GCT GGA TGC TCT CAT CTG-3′; IL-6, 5′-GCT ACC AAA CTG GAT ATA ATC GGA-3′ and 5′-CCA GGT AGC TAT GGT ACT CCA GAA-3′; macrophage inflammatory protein (MIP)-2, 5′-AAA ATC ATC CAA AAG ATA CTG AAC AA-3′ and 5′-CTT TGG TTC TTC CGT TGA GG-3′; IFN-γ, 5′-CGA CTC CTT TTC CGC TTC CTG AG-3′ and 5′-TGA ACG CTA CAC ACT GCA TCT TGG-3′; TNF, 5′-GCC ATG AGG TCC ACC ACC CTG-3′ and 5′-CTA CTG GCG CTG CCA AGG CTG T-3′; glyceraldehyde-3-phosphate dehydrogenase (G3PDH), 5′-CTA CTG GCG CTG CCA AGG CAG T-3′ and 5′-GCC ATG AGG TCC ACC ACC CTG-3′, respectively. Real time PCR was performed with QuantiTect SYBER green PCR kit (Qiagen, Venio, Netherlands) using an ABI7500 real-time PCR system and 7500 system SDS software (Applied Biosystems, Foster City, CA). mRNA was shown as the relative amount normalized to that of G3PDH.

### Small Interfering (si) RNA Transfection

MOC1 cells were transfected by lipofection (Lipofectamine RNAi MAX, Invitrogen, Carlsbad, CA) with the siRNA oligomers against either mouse MLCK (Mm Mylk 9988, Sigma), TNFR1 (MSS212008), TNFR2 (MSS238548) or non-targeting control (12935-112, Invitrogen) and incubated at a density of 5×10^4^ cells/ml in OPTI-MEM (Invitrogen) for 48 h.

### Transmission Electron Microscopy (TEM)

TEM was performed as previously described [20]. Briefly, colonic tissues from AOM/DSS-treated mice or MOC1 cells that were seeded on the Cell Disk® (Sumitomo, Tokyo, Japan) were fixed with 2.5% glutaraldehyde in 0.1 M phosphate buffer (PB) for 2 h. The samples were washed with 0.1 M PB, post-fixed in 1.0% OsO_4_ buffered with 0.1 M PB for 2 h, dehydrated in a graded series of ethanol and embedded in Epon-812. Semi-thin sections were cut at 1 µm and stained with toluidine blue. Ultrathin sections, 90 nm, were collected on copper grids, double-stained with uranyl acetate and lead citrate, and then observed using transmission electron microscopy (H-7100, Hitachi, Hitachinaka, Japan).

### Statistical Analysis

The results are expressed as the mean ± standard error of the mean (SEM). Statistical significance was determined using non-parametric Mann-Whitney *U*-test, and differences were considered to be statistically significant with p<0.05.

## Results

### Secretion of Cytokines that Support Tumor Growth is Associated with Epithelial MLCK Expression in the CAC Model

**Figure 1:**
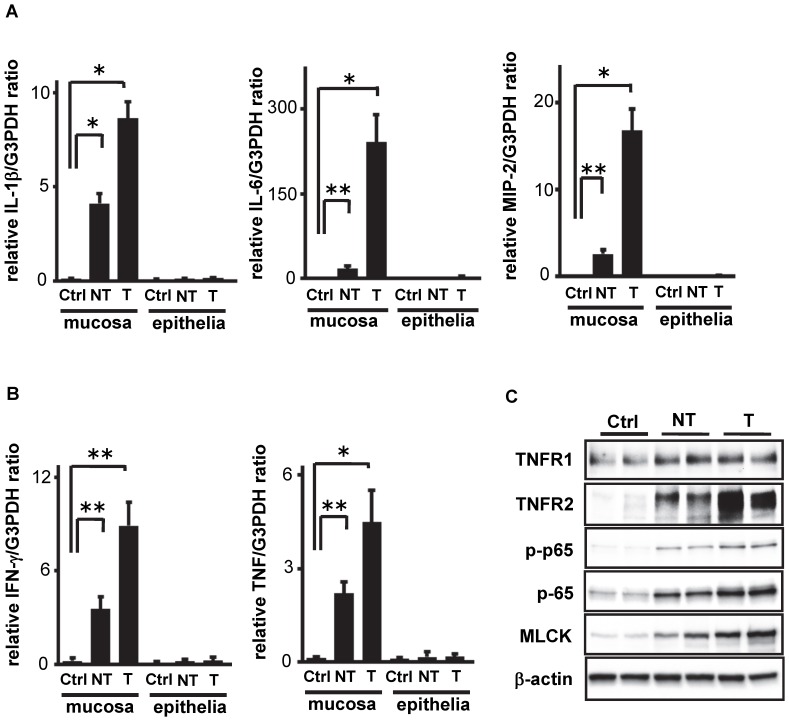
Pro-tumorigenic cytokines are increased in inflamed colonic mucosa, and further in tumors in AOM/DSS model. (A and B) Relative mRNA expressions for IL-1β, IL-6, MIP-2 (A), IFN-γ and TNF (B) in colonic mucosal tissues (mucosa) or isolated epithelial cells (epithelia) from either non-treated control (Ctrl), non-tumor (NT) or tumor tissues (T) in AOM/DSS treated mice are shown. Mucosal tissue samples including the epithelia and lamina propria were mechanically stripped from the whole colon to eliminate the muscularis layer, and RNA was then isolated. Mucosal tissue samples were also dissolved with 0.5 µM EDTA for 15 min, washed with PBS twice, subjected to density gradient centrifugation to isolate epithelial cells, and applied for RNA extraction. Each mRNA level was quantified by q-PCR and normalized to the level of G3PDH (n = 3). *; p<0.01, **; p<0.05. (C) Protein lysates of isolated epithelial cells from non-treated control (Ctrl), non-tumor (NT) or tumor tissues (T) were subjected to Western blotting with either anti-TNFR1, anti-TNFR2, anti-p-p65, anti-p65, anti-MLCK, or anti-β-actin Abs, respectively.

We previously observed that cytokines such as IL-1β, IL-6 and MIP-2 were up-regulated in an animal model of colitis [6]. This suggested that these factors may be essential for tumor development since each had been reported to support tumor growth in such model [21], [22], [23], [24]. However, it was unclear if these cytokines could be substantially up-regulated in the setting of CAC. In addition, it was also unclear if such elevated cytokines were mainly produced from the epithelia, since intestinal epithelial cells had been reported to be capable of expressing these molecules [25], [26], [27], [28]. Therefore we first assessed the expression levels of these cytokines in the mucosal tissues and the isolated epithelial cells in an animal model of CAC. Wild type C57BL/6 mice were administered AOM, then treated three times with DSS to induce chronic colitis and CAC. As seen in Fig. 1A, q-PCR revealed significant up-regulation of IL-1β, IL-6 and MIP-2 in the inflamed colonic tissues when compared to the control mice. Associated with this, significant up-regulations of pro-inflammatory cytokines, such as IFN-γ and TNF, were also observed as seen in Fig. 1B. In addition, the expressions of all of these cytokines were further up-regulated in the area of tumors compared to non-tumor area. Moreover, q-PCR also indicated that these cytokines were not much expressed by the epithelial cells (Fig. 1A and B). These results imply that such increase of these cytokines are presumably produced by infiltrating cells in the tissues such as granulocytes and macrophages and may be required for the progression of CAC.

Given these results, we hypothesized that the increased pro-tumorigenic cytokines produced by the infiltrating cells in the inflamed tissues may be associated with the disrupted epithelial tight junctions (TJ), which results in bacterial translocation into the lamina propria. In addition, we have also observed that TNFR2 expression was specifically up-regulated in the inflamed epithelia [6]. In this regard, it has been reported by Wang et al. that the up-regulated MLCK in human intestinal cells is required for TNF-induced collapse of epithelial TJ by TNFR2 signaling [11], [29]. Moreover, it has been reported by two groups that MLCK promoter activity is mediated by NF-κB activation [30], [31], [32]. Therefore, we addressed the epithelial MLCK expression in the CAC model. As seen in Fig. 1C, Western blotting revealed up-regulated MLCK expression in the epithelia from non-tumor area, and further up-regulation in that of tumor, in association with the up-regulated TNFR2 and p65. However, up-regulation of TNFR1 was not remarkable compared to that of TNFR2. These results indicate that the development of CAC may be associated with the disrupted TJ and elevated pro-tumorigenic cytokines, which are essentially induced by TNFR2 signaling and MLCK expression in the epithelia.

### MLCK Expression in the Epithelial Cells is Up-regulated by the Presence of Pro-inflammatory Cytokines in vitro

**Figure 2:**
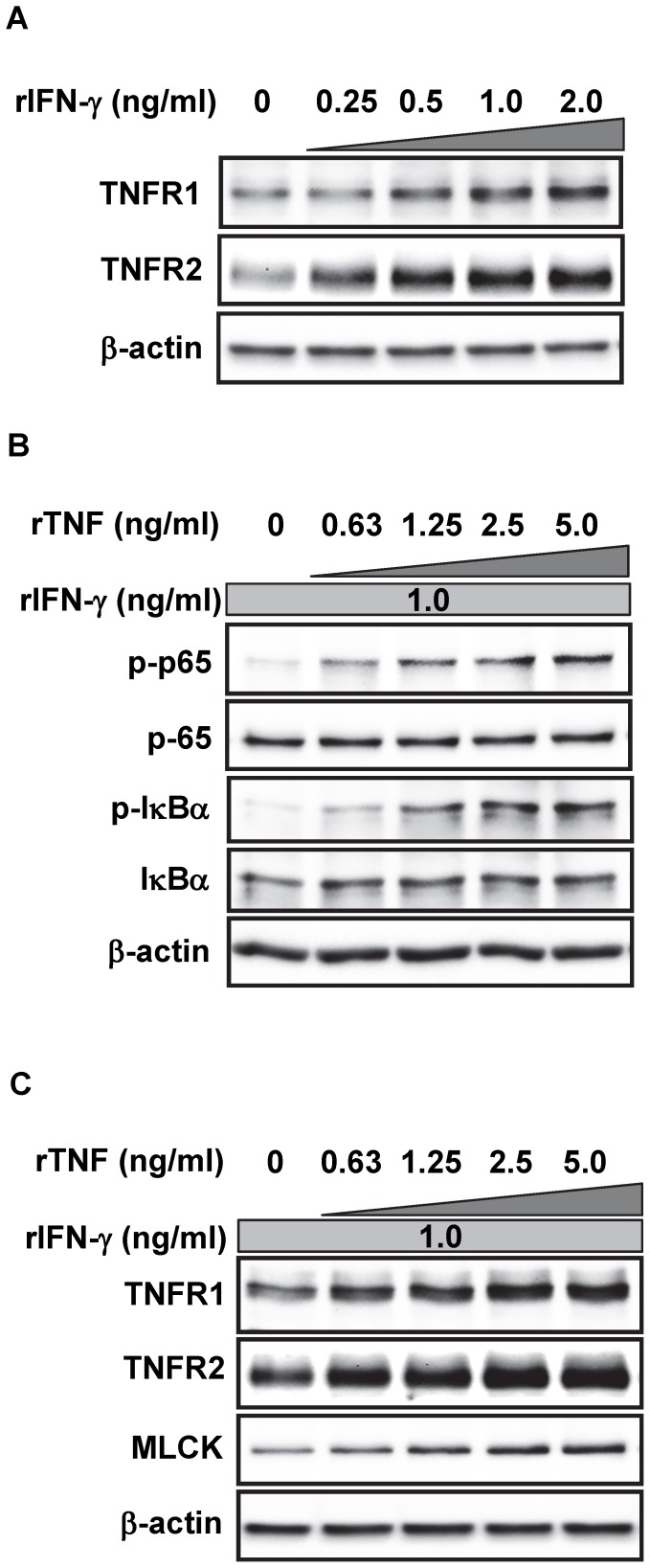
MLCK is up-regulated in the IFN-γ and TNF-stimulated MOC1 cells in association with NF-κB activation. (A) Protein lysates from MOC1 cells stimulated with the indicated concentration of rIFN-γ were subjected to Western blotting with either anti-TNFR1, anti-TNFR2 or anti-β-actin Abs. Representative data at 36 h after rIFN-γ stimulation are shown. (B) MOC1 cells were incubated with 1.0 ng/ml of rIFN-γ for 24 h, followed by the stimulation with the indicated concentration of rTNF, and subjected to Western blotting with either anti-p-p65, anti-p65, anti-p-IκBα, anti-IκBα or anti-β-actin Abs. Representative data at 5 min after rTNF stimulation are shown. (C) MOC1 cells were treated with 1.0 ng/ml of rIFN-γ for 24 h followed by the stimulation with the indicated concentration of rTNF and were subjected to Western blotting with anti-TNFR1, anti-TNFR2, anti-MLCK or anti-β-actin Abs. Representative data at 12 h after rTNF stimulation are shown.

Given the up-regulated TNFR2 and MLCK expressions in the epithelial cells associated with pro-tumorigenic and pro-inflammatory cytokines expressions in the mucosal tissues from chronic colitis and CAC model, it was surmised that each of these complicated phenomena may be associated with the mechanisms by which tumorigenesis is induced in the context of IBD. Especially, previous reports suggested that stimulation of human intestinal epithelial cell lines with IFN-γ results in the regulation of TNF receptors [29], [33], [34]. Therefore, a murine colonic epithelial cell line (MOC1) derived from colonic epithelial cell [15] was used to study the specific roles of TNFR2 and MLCK expressions in the epithelial cells in the context of inflammation. MOC1 cells were cultured in the presence of pro-inflammatory cytokines, rIFN-γ and rTNF. As seen in Fig. 2A, the up-regulations of TNFR1 and 2 were induced by rIFN-γ alone, and this is consistent with previous observations with human T84 and Caco2 cells [29], [33], [34]. However, the increased TNFR2 level was more remarkable compared to that of TNFR1, and such TNFR2 expression in MOC1 cells was maximal at the concentration of 1.0 ng/ml rIFN-γ (Fig. 2A).

Next, MOC1 cells were stimulated with different concentrations of rTNF together with rIFN-γ at 1.0 ng/ml. Western blotting showed that phosphorylations of p65 and IκBα were induced in these cells by the presence of rTNF in a dose-dependent manner (Fig. 2B). Moreover, the expressions of MLCK as well as TNFR2, but not much of TNFR1, were further up-regulated by the addition of rTNF in a dose-dependent fashion in collaboration with constant concentration of rIFN-γ, and these expressions were maximal at 2.5 ng/ml of rTNF (Fig. 2C). These results suggest that the pro-inflammatory cytokines, especially TNF, may be required for the induction of MLCK up-regulation via TNFR2 signaling in the colonic epithelial cells.

### MLCK Expression and Disrupted Intercellular Junctions are Induced by Up-regulated TNFR2, but not TNFR1

**Figure 3:**
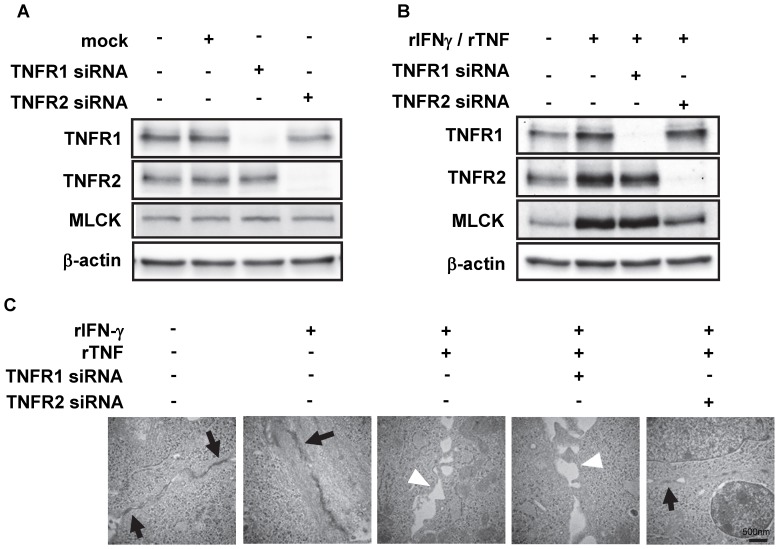
TNFR2-silencing in MOC1 cells diminishes TNF-induced MLCK up-regulation and disrupted epithelial tight junctions. (A) Protein lysates from MOC1 cells, which were transfected with either mock-, TNFR1- or TNFR2-targeting siRNA 36 h prior without rIFN-γ or rTNF stimulation, were subjected to Western blotting with either anti-TNFR1, anti-TNFR2, anti-MLCK or anti-β-actin Abs. (B) MOC1 cells were transfected with either mock-, TNFR1- or TNFR2-targeting siRNA, and then pre-incubated with or without 1.0 ng/ml rIFN-γ for 24 h prior to add 2.5 ng/ml rTNF for 12 h. Protein lysates from these cells were subjected to Western blotting with either anti-TNFR1, anti-TNFR2, anti-MLCK or anti-β-actin Abs. (C) MOC1 cells were transfected with either mock-, TNFR1- or TNFR2-targeting siRNA for 36 h with or without rIFN-γ and/or rTNF stimulation as indicated. Representative electron microscopic images from three experiments (n = 10) are shown. Arrows indicate fine intercellular junctional complexes including tight junctions, and arrow heads indicate disrupted intercellular junctional complexes. Scale bar indicates 500 nm.

Given the observation of up-regulated MLCK expression in association with the up-regulated TNFR2 expression in MOC1 cells, we next pursued the specific linkage between TNFR2 and MLCK in vitro. To do so, gene expressions of either TNFR1 or TNFR2 in MOC1 cells were silenced by transfection with specific siRNAs. We first confirmed the efficacies of siRNA oligomers against either TNFR1 or TNFR2 in these cells. As expected, knocked-down expressions of either TNFR1 or TNFR2 were observed with each specific siRNA (Fig. 3A). It should be noted that the endogenous expression of MLCK in MOC1 cells was not affected by the silencing of either TNFR1 or TNFR2 (Fig. 3A). We next assessed the effect of each silencing on the induction of MLCK up-regulation by pro-inflammatory cytokines in these cells. As seen in Fig. 3B, MLCK expression was up-regulated by stimulation with rIFN-γ and rTNF, and this is consistent with the results seen in Fig. 2C. In addition, the up-regulated MLCK expression in the presence of rIFN-γ and rTNF was not affected by the knocking-down of TNFR1 expression. However, such up-regulation of MLCK was remarkably suppressed by TNFR2 silencing (Fig. 3B). These results indicate that up-regulation of MLCK is specifically induced by TNFR2 signaling in the epithelial cells.

It was still unclear whether such up-regulated MLCK expression substantially affects the intestinal epithelial cells. Therefore, we next studied the morphology of MOC1 cells in the presence of rIFN-γ and rTNF. As seen in Fig. 3C, intercellular junctional complexes of these cells with intact TJ were observed using transmission electron microscopies (TEM) regardless of single stimulation with rIFN-γ. When stimulated with both rIFN-γ and rTNF, MOC1 cells showed collapsed intercellular junctional complexes with disappeared TJ, and the silencing of TNFR1 expression did not affect such features. However, the silencing of TNFR2 expression improved such disrupted intercellular junctions (Fig. 3C). Similar results were also observed using immunofluorescence microscopic studies (IFM) with anti-ZO1 pAb under confocal microscopies (data not shown). Taken together, these results suggest that the disrupted TJ among intestinal epithelial cells may be induced by TNFR2 signaling via MLCK up-regulation.

### The Blockade of TNF and the Suppression of MLCK Abrogate Disruption of Epithelial TJ in vitro

**Figure 4:**
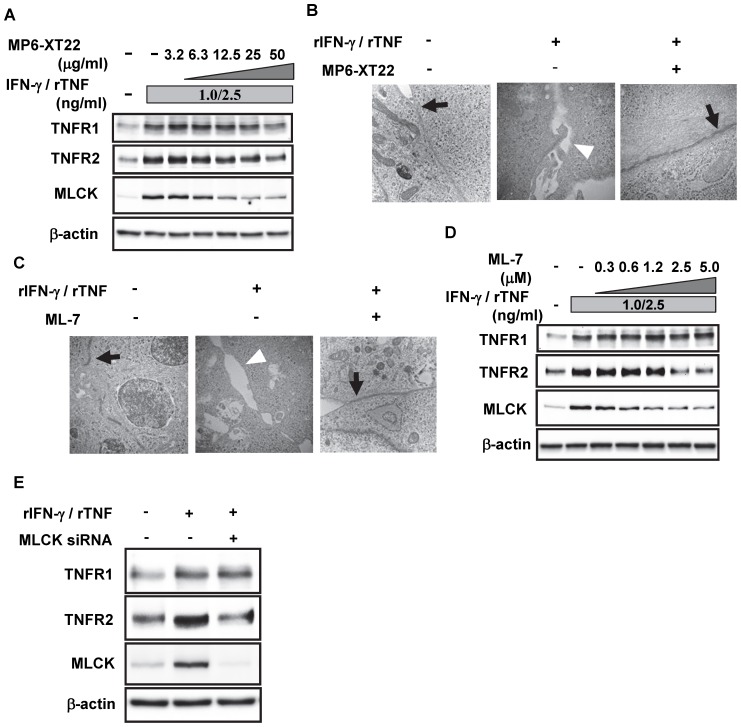
Suppressed MLCK functions may restore the TNF-induced disruption of intercellular junctions among MOC1 cells. (A) MOC1 cells were incubated with 1 ng/ml rIFN-γ and 2.5 ng/mL rTNF in the presence of indicated concentration of MP6-XT22. Protein lysates from these cells were subjected to Western blotting with either anti-TNFR1, anti-TNFR2, anti-MLCK, or anti-β-actin Abs. (B) MOC1 cells were incubated with rIFN-γ and rTNF in the presence or absence of 25 µg/ml MP6-XT22. Representative electron microscopic images from three experiments (n = 3) are shown. Arrows indicate fine tight junctions, and arrow heads indicate collapsed intercellular junctional complexes. (C) MOC1 cells were stimulated with rIFN-γ and rTNF, as described above, in the presence or absence of 2.5 µM ML-7. Representative electron microscopic images from three experiments (n = 3) are shown. Arrows indicate fine tight junctions and arrow heads indicate collapsed intercellular junctional complexes. (D) MOC1 cells were stimulated with rIFN-γ and rTNF, as described above, in the presence of the indicated concentration of ML-7. Protein lysates from these cells were subjected to Western blotting with either anti-TNFR1, anti-TNFR2, anti-MLCK or anti-β-actin Abs. (E) MOC1 cells were transfected with MLCK-targeting siRNA and stimulated with rIFN-γ for 24 h prior to adding rTNF for 12 h. Protein lysates from these cells were then subjected to Western blotting with either anti-TNFR1, anti-TNFR2, anti-MLCK or anti-β-actin Abs, respectively.

Given the TNFR2 signaling-dependent up-regulation of MLCK, we next assessed the effect of blocking TNF signaling in the epithelial cells on the TJ, which may be disrupted by up-regulation of MLCK. MOC1 cells were cultured in the presence of rIFN-γ to induce TNFR2 expression followed by addition of rTNF. As seen in Fig. 4A, the up-regulated MLCK expression was observed in these cells in association with remarkable TNFR2 up-regulation. In addition, such up-regulations of TNFR2 and MLCK were abrogated by the presence of MP6-XT22, a mAb against to TNF, in a dose-dependent fashion (Fig. 4A). It should be noted that slightly up-regulated TNFR1 expression by rTNF stimulation was also suppressed in the presence of MP6-XT22. Given the abrogation of MLCK up-regulation by MP6-XT22, we next studied the intercellular junctions among MOC1 cells under this condition. IFM (data not shown) and TEM (Fig. 4B) studies showed restoration of TJ in the presence of MP6-XT22. These results suggest that TNF signaling in the colonic epithelia may induce disruption of TJ via up-regulated TNFR2 and MLCK expressions. Moreover, these results also suggest that neutralization of TNF may abrogate such TJ disruption.

To confirm TJ disruption by MLCK up-regulation, rIFN-γ/rTNF-stimulated MOC1 cells were also incubated in the presence of ML-7, an MLCK inhibitor. As expected, the disturbed TJ, which was induced by the presence of rIFN-γ and rTNF, was restored by the inhibition of MLCK function (Fig. 4C). This result was originally considered to be the cause of suppressed MLCK function by ML-7. However, Western blotting showed that the up-regulated TNFR2 and MLCK expressions were interestingly suppressed by the addition of ML-7 in a dose-dependent fashion (Fig. 4D). It should be noted that the up-regulated TNFR1 expression induced by rIFN-γ and rTNF was not affected by the addition of ML-7. Given this result, MOC1 cells were also transfected with MLCK-specific siRNA. As seen in Fig. 4E, up-regulation of TNFR2 induced by rIFN-γ and rTNF was suppressed by silencing MLCK expression. Taken together, these results indicate that the disrupted TJ among intestinal epithelial cells induced by TNFR2 signaling may be restored by the suppression of MLCK function.

### Suppression of MLCK Prevents the Development of CAC in Association with Restored TJ and Diminished Pro-tumorigenic Cytokine Productions in vivo

**Figure 5:**
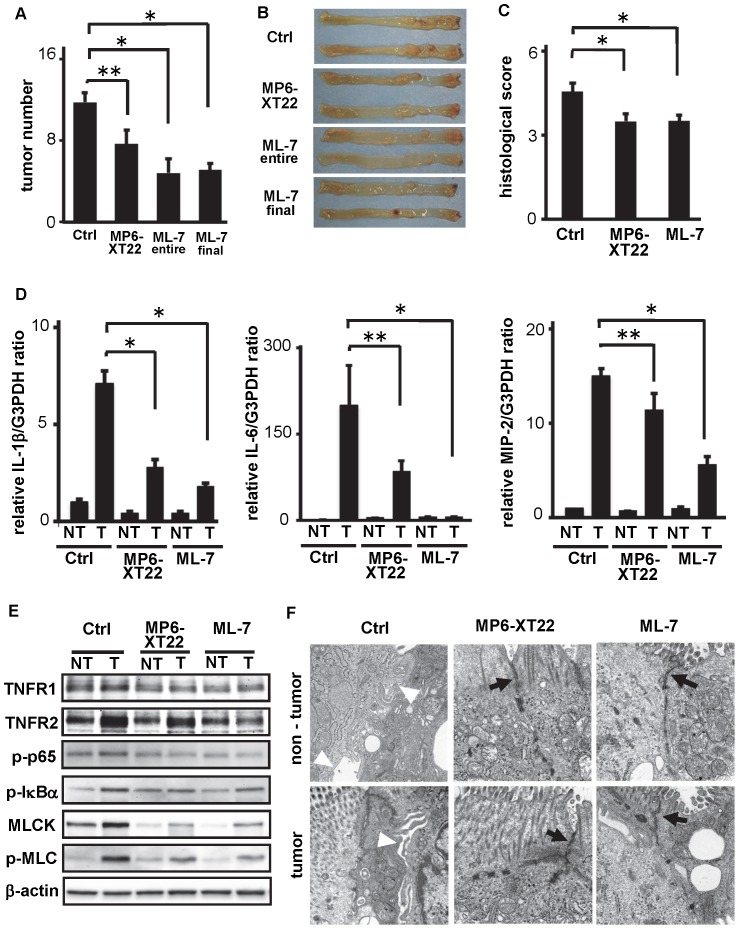
Treatment with ML-7, as well as with MP6-XT22, may reduce CAC development in AOM/DSS model. (A) Numbers of microscopically indicated tumors per colon in AOM/DSS-administered mice with either vehicle control (Ctrl), anti-TNF Ab (MP6-XT22), ML-7 treatment during the entire protocol (ML-7 entire), or ML-7 treatment at the final phase of protocol (ML-7 final) (n = 10) are shown. Tumors were determined under the microscope. Data are expressed as means ± SEM. *; p<0.01, **; p<0.05. (B) Macroscopic overview of representative colonic samples from each group is shown. (C) Histological assessment of colitis in mice treated with either control, MP6-XT22 or ML-7 (at the final phase) (n = 10). Data are expressed as means ± SEM. *; p<0.05. (D) Relative mRNA expressions for IL-1β, IL-6 MIP-2 and TNFR2 in inflamed mucosa from non-tumor or tumor tissues in mice treated with either control, MP6-XT22 or ML-7 (final phase) were analyzed. Each mRNA level was quantified by RT-PCR and normalized to the level of G3PDH (n = 3). *; p<0.01, **; p<0.05. (E) Protein lysates of the isolated epithelial cells from non-tumor or tumor tissues in mice administered with either control, MP6-XT22 or ML-7 (final phase) treatment were subjected to Western blotting with either anti-TNFR1, anti-TNFR2, anti-p-p65, anti-pIκBα, anti-MLCK, anti-p-MLC, or anti-β-actin Abs, respectively. (F) Electron microscopic images of representative non-tumor and tumor samples from either control, MP6-XT22 or ML-7 (final phase) treated group are shown (n = 10). Arrows indicate fine tight junction, and arrow heads indicate disrupted intercellular junctional complexes.

Given the disrupted TJ via TNFR2 signaling and MLCK up-regulation in vitro, we finally assessed the effect of MLCK suppression in the setting of CAC. Mice induced with CAC by AOM treatment and three cycles of DSS were injected with either vehicle control, MP6-XT22 or ML-7, and later euthanized to assess colonic inflammation and CAC development. As seen in Fig. 5A and B, the blockade of TNF in vivo, by weekly injection with MP6-XT22 throughout the entire experimental protocol, resulted in the reduction of CAC development. This result is consistent with our previous study [6]. Moreover, suppression of MLCK, by the injection with ML-7 during the entire protocol resulted in even more reduction of CAC development (‘ML-7 entire’ in Fig. 5A and B). In addition, administration of ML-7 at the recovering phase after the last DSS administration surprisingly revealed almost same degree of suppression on CAC development (‘ML-7 final’ in Fig. 5A and B). These results were relatively associated with the histological assessment of colitis in these groups, although the significances of colitis suppression with either administration of MP6-XT22 or ML-7 were not remarkable compared to that of CAC suppression (Fig. 5C). Interestingly, q-PCR revealed that the expression levels of IL-1β, IL-6 and MIP-2 (Fig. 5D) as well as IFN-γ and TNF (data not shown) in the colonic tissue, especially in the tumor area, were suppressed by the treatment with ML-7 as well as with MP6-XT22 (Fig. 5D). In addition, the MLCK expression and MLC phosphorylation in the isolated epithelial cells from the non-tumor and tumor tissues were remarkably suppressed by the treatment with MP6-XT22 or ML-7 (Fig. 5E). It should be noted that the epithelia isolated from tumor area in each groups showed relatively higher expressions of TNFR2, p-p65, p-IκBα, MLCK and p-MLC when compared to that in non-tumor epithelia. These results are consistent with the observations of the intercellular junctional complexes among cells under TEM, since chronic inflammation in the control group induced disruption of epithelial TJ, and treatments via both TNF blockade and MLCK suppression resulted in the restoration of the disrupted TJ in the non-tumor and tumor areas and in association with reduced CAC (Fig. 5F). These results indicate that the suppression of MLCK function may contribute to reduce CAC development via restoration of the disrupted TJ.

## Discussion

The etiology of IBD is considered to be associated with both epithelial permeability and dysregulated immune responses to luminal contents which include antigens derived from commensal bacteria in the gut. Regarding immune dysregulation, in patients with Crohn’s disease for example, excessive amount of Th1 and Th17 cytokines, such as IFN-γ and IL-17 respectively, are secreted predominantly by the infiltrating CD4^+^ effecter T cells in the intestinal lamina propria [19], [35], [36]. TNF, a pro-inflammatory cytokine produced not only by such dysregulated effecter T cells but also by macrophages and granulocytes infiltrating the inflamed intestinal tissues, is involved in the dysregulated adoptive immune responses in CD and possibly UC as well. Current therapeutic approaches in neutralizing TNF using either chimeric or humanized Abs have provided some effective therapies in the management of CD [1], [37], [38] and to some extent UC as well [39]. Our efforts in the study of pathogenesis of IBD have shown that TNF is expressed mainly by F4/80^+^ macrophages rather than CD4^+^ T cells in the DSS colitis model [6].

It is known that myeloid cell-derived macrophages and granulocytes are also capable of expressing other cytokines such as IL-1β, IL-6 and MIP-2, a homolog of IL-8 in humans. These cytokines can also induce pro-tumorigenic activities such as angiogenesis and tumor proliferation [21], [22], [23], [24], but at the same time, these cytokine may also be expressed by intestinal epithelial cells [25], [26], [27], [28]. In fact, we have observed in previous and current studies that the expression levels of these cytokines in colonic tissues are up-regulated in DSS colitis and further up-regulated in tumors. CAC development was suppressed by the treatment with either blocking anti-TNF mAb or MLCK inhibitor in association with reduced cytokine productions mentioned above. However, such cytokine expressions from the isolated colonic epithelial cells were not significant when compared to that of the entire colonic mucosa. Therefore, pro-tumorigenic cytokines most likely derived from the lamina propria where most macrophages reside. Others had also shown that NF-κB activation in the myeloid cells is critical for the induction of CAC [5], and that NF-κB activation in myeloid cells can be induced by TNF [40].

DSS colitis is a commonly utilized murine model of IBD where a single administration of DSS can lead to acute colitis. In addition, three cycles of DSS administration can lead to chronic inflammation in the colon. Furthermore, AOM administration preceding three cycles of DSS treatment can lead to epithelial carcinogenesis and is a model of CAC [41]. By using this CAC model, Greten and colleagues showed that epithelial NF-κB activation is critical for the development of CAC [5]. However, the roles and mechanisms of epithelial NF-κB activation leading to CAC development had not previously been rigorously studied. In our previous and current studies, we demonstrated epithelial NF-κB activation in association with up-regulated TNF production by macrophages infiltrating the colonic lamina propria in the same CAC model. In addition, we showed that epithelial NF-κB activation was abrogated by neutralizing TNF with a specific mAb leading to the suppression of CAC [6]. These results suggest that the NF-κB pathway in the epithelial cells is mainly activated by the up-regulated TNF production. Previous studies by others had suggested that insufficient immunological homeostasis due to TNF production in IBD was caused by sequential activation of macrophages and neutrophils dominantly expressing TNFR1 (TNFRSF1a/p55) in the inflamed tissue [40]. However, we and other researchers had observed that intestinal epithelial cells express both TNFR1 and TNFR2 (TNFRSF1b/p75) [6], [42]. There is spontaneous TNFR2 up-regulation in the epithelial cells in the setting of in vitro and in vivo inflammatory conditions [6], [43]. Increased expression of TNFR2 in the epithelia was associated with CAC development [6]. These results suggest that TNF-induced epithelial NF-κB activation is associated with CAC development.

Given the results of up-regulated TNFR2 expression and NF-κB activation in the inflamed epithelial cells, we hypothesized that this may somehow be associated with reducing the recruitment of the death domains, such as FADD and TRADD, which are involved in the caspase-dependent pathway of TNFR1 signaling, so that the epithelial cells may escape apoptosis. However, the role of the epithelial NF-κB activation via TNFR2 signaling associated with CAC induction was not known previously. Therefore, we investigated whether increased expression of TNFR2 in this model leads to CAC. Using the same CAC model, TNFR2 deficient mice were used. However, CAC development was not possible due to high mortality with DSS treatment which had also been observed previously [6]. Therefore, we have utilized MOC1, which is a murine epithelial cell line derived from non-cancerous colonic epithelia transformed with SV40 large T antigen [15]. This cell line likely possess more physiological functions compared to other cell lines derived from murine colon cancer such as CT26 cells [44]. Furthermore, MOC1 cells can form confluent monolayers with epithelial TJ. Such characters of MOC1 cells allowed us to assess the collapsed TJ associated with MLCK up-regulation, because it was difficult to observe such morphologies with CT26 cells, which does not show either MLCK up-regulation or fine monolayers required for TJ observation.

It had been shown that MLCK in human intestinal cells leads to TNF-induced collapse of epithelial TJ via TNFR2 signaling [29] and that MLCK promoter activity is mediated by NF-κB [30], [31], [32]. Therefore, in our study, MOC1 cells were transfected with the TNRF2 siRNA to determine MLCK activities in the presence of rTNF. This resulted in the abrogation of TNF-induced TJ disruption associated with suppressed MLCK up-regulation (Fig. 3B and C), and these results are consistent with previous reports [11], [29]. Moreover, we also observed that the TJ remained intact with anti-TNF treatment as well as inhibiting MLCK activity (Fig. 4B and C). In our current study, we also observed that TNFR1 expression in MOC1 cells was slightly up-regulated by addition of rTNF, and such up-regulated TNFR1 was suppressed by the presence of MP6-XT22. These results imply that presence of TNF may induce both up-regulations of TNFR1 and TNFR2 in inflamed epithelial cells as reported by Mizoguchi et al [42], [43], suggesting that NF-κB activation in the epithelial cells we observed may be caused by both signaling. However, the degree of TNFR2 up-regulation was remarkably more than that of TNFR1 (Fig. 2A, 2C, 4A and 4D). Moreover, TNFR1 silencing failed to restore irritated intercellular junctions in association with not down-regulated MLCK expression (Fig. 3B and C). These results indicate that TNFR2 signaling is the key factor for MLCK-mediated epithelial permeability, as consistent with the observations by Wang et al [29]. These findings may also imply an important mechanism by which NF-κB pathway via TNFR2 signaling is regulated in the colonic epithelial cells in the setting of carcinogenesis. Thus, one potential mechanism by which TNFR2 expression in intestinal epithelial cells is associated with CAC development may be that the epithelial barrier dysfunction induced by MLCK activation via TNFR2 signaling leads to translocation of luminal bacteria into the lamina propria. Such epithelial permeability results in the stimulation of macrophages and granulocytes in mucosal tissue followed by cytokine production such as IL-1β, IL-6 and MIP-2 that are required for epithelial proliferation and tumor development. On the other hand, it should be noted that the impact of such pro-tumorigenic cytokines production, which is caused by disrupted TJ, on the severity of DSS colitis is controversial. Some recent studies have suggested that suppression of epithelial permeability by administration of MLCK inhibitors remarkably ameliorate DSS colitis [12], [13]. Our current results also suggest that colitis was suppressed by ML-7 treatment, however, the impact of such treatment on the suppression of colitis was not remarkably effective compared to that on the suppression of CAC development. One reason for the different observations may be that the severity of colitis in our studies was relatively mild compared to the others, and thus, we did not observe distinct abrogation of colitis by MLCK inhibition. In addition, another recent report showed that MLCK deficient mice revealed worse DSS colitis compared to wild type [14]. These discrepant observations by others and ours suggest that another reason may be contributed by environmental factors such as the different microflora at the animal facilities that may induce different cytokine profiles.

In our current study, we observed that IFN-γ and TNF-induced up-regulation of TNFR2, and possibly MLCK, in the epithelial cells were specifically suppressed by ML-7 treatment (Fig. 4D). Up-regulation of TNFR2, but not TNFR1, was also suppressed by silencing MLCK expression (Fig. 4E). These results suggest that TJ disruption, induced by MLCK expression and MLC phosphorylation in the epithelial cells, may result in further up-regulation of TNFR2 expression in vitro. This in turn causes a vicious circle on the epithelial permeability, and such condition may promote tumor growth in the context of inflammation. Such impact of the relationship between TNFR2 and MLCK on CAC development is also suggested by the fact that the administration of ML-7, even at the final phase of CAC induction, resulted in the remarkable reduction of tumor in mice (Fig. 5A–F). In fact, ML-7 injection after final cycle of DSS treatment seemed to result in almost the same effect as the injection during the entire protocol in terms of reduction of tumor number, shown in Fig. 5A. It is suggested that colonic epithelial cells have undergone enough inflammation to initiate carcinogenesis until mice were injected with ML-7 at the final stage. One potential interpretation for this observation would be that undetectable microscopic tumors or aberrant crypt foci, which had not developed yet in the colonic epithelia, may still exist in mice treated with ML-7 only at the final phase. However, it is still suggested that the inhibition of MLCK function is absolutely effective at least for the suppression of tumor progression. In addition, we have examined whether such MLCK expression also induce up-regulation of endogenous IFN-γ and/or TNF expressions in MOC1 cells. However, neither expressions were affected regardless of MLCK expression (data not shown). Therefore, another mechanism may be involved in TNFR2 up-regulation in the irritated epithelial cells. Furthermore, our current study suggests that MLCK expression, which may be induced by TNFR2 signaling in the intestinal epithelial cells, can be a therapeutic target for the maintenance of continuous inflammation and prevention of CAC development in the setting of IBD.

Here, we demonstrate TNFR2-mediated regulation of CAC development, which is due to tumorigenic cytokines induced by MLCK-induced disruption of the TJ. This is an important mechanism that helps us understand the regulation of mucosal immune response as well as IBD- associated epithelial tumorigenesis.
